# Cross-situational word learning is both implicit and strategic

**DOI:** 10.3389/fpsyg.2014.00588

**Published:** 2014-06-13

**Authors:** George Kachergis, Chen Yu, Richard M. Shiffrin

**Affiliations:** ^1^Department of Psychology, Leiden Institute for Brain and Cognition, Leiden UniversityLeiden, Netherlands; ^2^Cognitive Science Program, Department of Psychological and Brain Sciences, Indiana UniversityBloomington, IN, USA

**Keywords:** implicit learning, language acquisition, cross-situational word learning, automaticity, statistical learning

## Abstract

For decades, implicit learning researchers have examined a variety of cognitive tasks in which people seem to automatically extract structure from the environment. Similarly, recent statistical learning studies have shown that people can learn word-object mappings from the repeated co-occurrence of words and objects in individually ambiguous situations. In light of this, the goal of the present paper is to investigate whether adult cross-situational learners require an explicit effort to learn word-object mappings, or if it may take place incidentally, only requiring attention to the stimuli. In two implicit learning experiments with incidental tasks directing participants' attention to different aspects of the stimuli, we found evidence of learning, suggesting that cross-situational learning mechanisms can operate incidentally, without explicit effort. However, performance was superior under explicit study instructions, indicating that strategic processes also play a role. Moreover, performance under instruction to learn word meanings did not differ from performance at counting co-occurrences, which may indicate these tasks engage similar strategies.

## 1. Introduction

Humans have a remarkable capacity to adapt to the regularities in our environment, and our everyday actions—from navigating highways to navigating conversations—bear testament to this ability. We often seem to adapt without overt effort or even awareness of either the regularity, or of our changing behavior. Dubbed *implicit learning* (Reber, 1967), this automatic adjustment to the world is studied using a variety of experimental paradigms with different measures of learning. For example, in artificial grammar learning (AGL) experiments, originated by Reber (1967), participants first observe strings of letters generated from a grammar without being told the rules of the grammar (or that such rules exist), and learning is measured by their accuracy on grammaticality judgments for a set of new test strings. Despite being mostly unable to enumerate the rules used to generate grammatical strings, participants had above-chance accuracy on the judgments. This is sometimes construed as evidence that participants have absorbed the underlying generative grammar, although modeling work suggests that recognition memory is sufficient for making these judgments via global similarity comparisons (Jamieson and Mewhort, 2009).

Another class of tasks measures temporally-extended sequence learning, exemplified by the serial reaction time (SRT) task (Nissen and Bullemer, 1987), in which participants are asked to press one of four keys when the corresponding light comes on. After each keypress and a 500 ms interval, a subsequent light illuminates. In one condition, participants produced keypresses for a random sequence of lights, while in the other condition subjects unwittingly repeating the same sequence of 10 keypresses dozens of times. These latter participants showed greater increases in speed than participants given the random sequences. As in AGL experiments, at debriefing participants were generally unaware of learning, leading many to consider it implicit learning (see Shanks, 2005 for a review). Recent mousetracking results show that as learning proceeds, trajectories begin to bend in anticipation of the next response location, demonstrating that participants predict the next response and adapt their movements, much like neighboring phonemes in a word are co-articulated (Kachergis et al., 2014a). Implicit learning has long been assumed to be important to language learning (for a review, see Romberg and Saffran, 2010), and indeed knowledge of the sequential structure of language (that is, word predictability) has been shown to correlate with the implicit learning ability to detect sequential structure in visual stimuli (Conway et al., 2010). Moreover, Kidd (2012) demonstrated that implicit learning ability on a SRT task in 4- to 6-year-old children was correlated with grammatical learning ability in a syntactic priming task. Thus, the various paradigms of implicit learning are relevant to language acquisition, and these areas would benefit from further cross-pollination.

Indeed, the recent literature on statistical learning has in large part focused on how infants learn language (though see also Fiser, 2001; Turk-Browne et al., 2005), investigating problems such as segmenting continuous streams of speech into words, and learning which words refer to which objects. Whether or not the infant is performing these tasks explicitly or implicitly, the motivations and predictions made by statistical learning researchers overlaps remarkably with those in implicit learning (Cleeremans et al., 1998; Perruchet and Pacton, 2006). The seminal work on statistical learning (Saffran et al., 1996) demonstrated that infants are sensitive to statistical regularities in a continuous stream of an audible artificial language, enabling them to distinguish probable syllable sequences (i.e., words) from improbable syllable sequences. Newport (2004) found that infants are also sensitive to temporally distal regularities, which weighs in favor of a more general statistical learning mechanism, rather than a simple mechanism for associating adjacent sounds. Saffran et al. (1997) found that children and adults incidentally learned to segment words from a continuous stream of speech while they were engaged with an computer-based illustration task. Multimodal studies have found that infants can acquire nouns via the repeated co-occurrence of words and their referents across situations containing multiple words and objects, which are thus separately ambiguous (Smith and Yu, 2008).

As in adult implicit learning studies, infant statistical learning studies present participants with structured training data but no explicit learning instructions, and find behavioral differences due to the statistical regularities in the training data. Like implicit learning, statistical learning is often thought to proceed automatically from basic mechanisms of learning and memory, without requiring intentional strategies. However, adults in statistical learning tasks are typically given instructions relating to the learning goal: e.g., “Learn which words go with which objects.” The goal of the current paper is to empirically investigate the automaticity of cross-situational statistical word learning in adults, who are typically given explicit instructions to learn the meaning of the words (e.g., Yu and Smith, 2007). In Experiment 1, we presented participants with a set of spoken words and visual objects with one-to-one mappings between them, but framed the task as one of recognition memory for individual stimuli, and not as one of learning word-object mappings. We then gave participants an unexpected test: for each of 54 word-object pairings, they were asked to indicate how often the word and object co-occurred. With their attention focused on memorizing individual words or visual objects, would participants unintentionally learn which words and objects co-occurred more frequently? In Experiment 2, we used a signal detection task as another incidental task to direct participants' attention to both auditory and visual streams, but again with no explicit instructions to learn word-object mappings. We then administered a surprise test to assess their knowledge of word-object mappings. In both experiments, after the initial implicit learning blocks, as a measure of their statistical learning capability for comparison, participants also completed blocks in which they were explicitly instructed to either count word-object co-occurrences, or simply to learn the meaning of the words.

We introduce the cross-situational learning paradigm below, and then discuss the possible learning mechanisms and potential contributions of the present implicit learning studies to advance our understanding of statistical learning. Aside from establishing whether cross-situational learning falls in the broader category of implicit learning, it is important to know if word learning is purely strategic in adults because some language acquisition research uses adults in lieu of infants, who cannot be explicitly instructed to do any particular task. If research on adults is capable of informing developmental language acquisition, we suggest it may be important for adult word learning to proceed at least in part automatically. In two implicit learning experiments with adult participants, we explore whether word learning is purely implicit, purely strategic, or a mixture. Finally, we conclude by summarizing the results from the two studies and discussing the connection between statistical and implicit learning.

In a typical version of cross-situational learning, adults are asked to learn which word goes with each object, and are then shown a series of training trials, each of which contains four objects (e.g., a sculpture) and four spoken pseudowords (e.g., “manu”). Because correct word-referent pairings are not indicated, learners can utilize only the repeated co-occurrence of words with their intended referents to learn across many trials. In a typical learning scenario (e.g., Yu and Smith, 2007), participants attempted to learn 18 pseudoword-object pairings from twenty-seven 12-second trials. This design allowed each stimulus (and hence each correct word-referent pairing) to be presented six times. In one form or another, the learning of a pairing involves the accumulation of word-object co-occurrence statistics across the training trials. Participants acquired, on average, 9 of the 18 pairs, as measured by a four-alternative forced choice (4AFC) referent test for each word.

**Figure 1:**
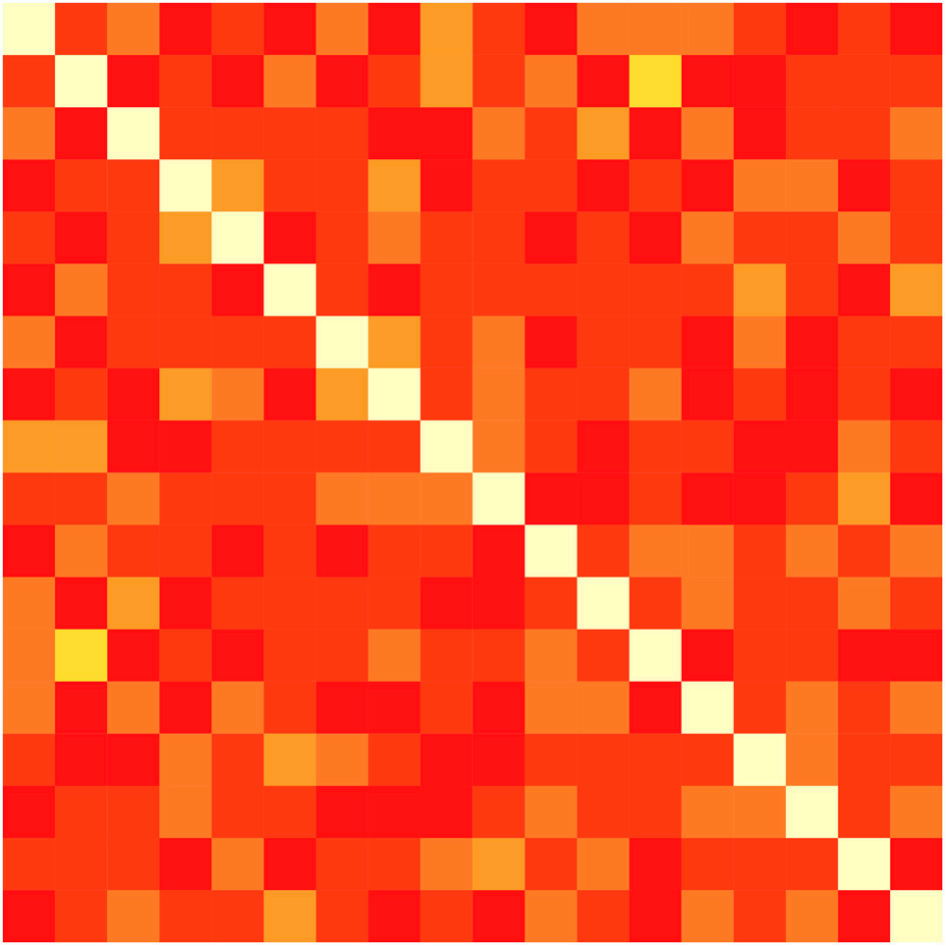
**Word × Referent matrix with the co-occurrences of each of the 18 words and objects accumulated across the 27 training trials used in each condition for both experiments**.

When each trial contains 16 possible word-referent associations, how might learning proceed? There are at least two distinct approaches that learners may apply. First, an ideal associative learner may maintain a word × object co-occurrence matrix *M*, incrementing the count in cell *M*_*w*,*o*_ whenever word *w* and object *o* appear together in a trial. Figure 1 shows such a matrix, which represents the training statistics used in the present study. At test, such a learner may choose the most frequently co-occurring referent for each word. Associative models typically approximate this co-occurrence matrix by strengthening a randomly sampled (perhaps according to current association strengths) subset of pairings on each trial. The association of spatiotemporally proximal stimuli could be carried out by automatic processes that require neither strategy nor intent to learn. Modern memory models such as REM (Shiffrin and Steyvers, 1997) even predict such associations by allowing feature values of nearby items to accidentally be recorded in an item's trace.

Another plausible learning approach is implemented in rule- and inference-based models (e.g., Siskind, 1996; Medina et al., 2011), which propose and store a number of hypothesized word-object pairings on each trial. Proposals may be made with respect to constraints such as mutual exclusivity, and hypothesized pairings may be confirmed if consistent evidence is presented later or removed from the lexicon if contradictory evidence is observed. This type of learning is more in accord with a deliberative, strategic learning process. If cross-situational learning is largely automatic, one may expect participants to have some knowledge of which words and objects frequently co-occurred during training, even when they were not explicitly trying to learn these relations. On the other hand, if cross-situational learning relies on more strategic, intentional inferences, then participants may perform much worse in such an incidental learning condition. Thus, the results from incidental learning tasks may shed light on the underlying learning mechanisms that learners use.

Finally, a variety of mixed strategies are possible, in which participants might roughly track co-occurrences and leverage this knowledge to intentionally select particular pairings for storage and testing. Indeed, in a study that presented a single word on each trial for immediate testing, with a varying number of objects (one of which was a target), participants were best modeled as using a mixed strategy on trials with high ambiguity (i.e., five or eight distractor objects; Smith et al., 2011). In this approximate cross-situational learning strategy, participants were assumed to pick the previously-picked referent if it is still present, and otherwise choose from the available referents with probability proportional to their co-occurrence frequency with the word. Thus, although participants are assumed to have a single explicit hypothesis, they form such hypotheses using background knowledge that reflects the statistical structure of the training trials. Smith et al. (2011) also found evidence that many participants used inferential guess-and-test, but only when the word was presented consecutively, and even then mostly when there were only two distractors.

In contrast, the current study presents a greater number of possible word-object mappings on each trial—four words and four objects—over a longer training period with no consecutive appearances of words or objects. Moreover, the current study does not test throughout training, which may encourage learners to make hypotheses, but only after training is finished. In particular, the present study will test participants' knowledge not only of the correct pairings (i.e., the diagonal cells of Figure 1) as is typically done, but also of the spurious word-object co-occurrences (non-diagonal cells) that appear during training—the sort of detailed and partial information that is stored by associative models (or an ideal learner), but typically not by rule-based models. We do this by asking participants to rate the strengths of co-occurring word-object pairings for both correct and incorrect pairings.

The testing paradigm allows us to both access participants' knowledge of spurious pairs and to compare that with what they know about correct pairs. Previous work has found evidence that people are sensitive to how often words and objects have co-occurred—even when a single object appears with a few words with differing frequency (Vouloumanos, 2008). However, Vouloumanos presented only a single word-object pair per trial, giving participants no choice as to which pairings to attend. In contrast, our paradigm offers 16 possible pairings per trial. Thus, the presence of four concurrent objects and four successive words per trial demands that participants modulate their attention, possibly forming stronger associations between particular words and objects, or perhaps attending only a subset of possible pairings. Thus, it is unclear how well participants' co-occurrence ratings will be correlated with actual stimuli co-occurrences in the explicit conditions, since inference-based learners may only track a lexicon of the most likely pairs (i.e., high co-occurrence stimuli), rather than a full matrix of associations.

## 2. Experiment 1

Every participant went through four blocks of training and testing in a fixed order. Training and testing in block 0 was structured differently than the remaining three. Participants were told that they would see multiple objects and hear multiple words on each trial, and that they should remember each object and word because their memory will be tested at the end. After the brief training period in block 0, they were given a recognition memory test: a single stimulus (word or object) was presented, and they were asked to label it old or new. The purpose of block 0 was to introduce the memory cover task to participants, demonstrating that individual stimuli would need to be recognized.

In block 1, participants were told again that they should remember each object and word for a subsequent memory test. However, after this training period, participants were given a surprise test of their knowledge of stimuli co-occurrences. Thus, the purpose of the *Memory* condition was to test incidental learning of word-object associations after studying individual stimuli for recognition. In block 2, participants were explicitly asked to remember how many times each word and object appeared together during training. They were not told what type of test to expect, but the co-occurrence rating test given was exactly the same as in block 1. This *Count Co-occurrences* condition directs subjects' attention to the conjunction of the words and objects rather than individual stimuli, providing one explicit comparison for the implicit Memory condition. In block 3, participants were simply asked to learn the meanings of the words—explicit learning instructions like those given in previous cross-situational word learning studies. This *Word Meanings* condition investigates whether word-learning strategies used by participants will significantly alter their behavior or performance as compared to the *Count Co-occurrences* condition. Note that while the fixed block order succeeds in obscuring the true objective of the study (i.e., how people learn word meanings) until the final block, it has the limitation that strategies induced by earlier instructions could carry over, or limited practice effects may be seen.

### 2.1. Materials and methods

#### 2.1.1. Subjects

Participants were 35 undergraduates at Indiana University who received course credit for participating. None had participated in other cross-situational experiments.

#### 2.1.2. Stimuli

Verbal stimuli were 72 computer-generated pseudowords that are phonotactically-probable in English (e.g., “bosa,” “manu,” and “stigson”), and were spoken by a monotone, synthetic female voice. Objects were 72 photos of uncommon, difficult-to-name objects (e.g., strange sculptures). Of these sets of objects and words, 54 were randomly assigned to three sets of 18 word-object pairings; one set for each study condition. The remaining 18 words and 18 objects were used for an initial recognition memory test. The set of stimuli that the words and objects were randomly selected and paired from for each participant is available online here: http://kachergis.com/downloads/stimuli.zip.

In block 0, each trial presented three unusual objects concurrently and three pseudowords heard in succession. Block 0's training consisted of only three 11-second trials, displaying nine unique words and objects once each. After these trials, participants were given a yes/no recognition test for each trained word and object, as well as nine new words and objects. On each test trial, a single stimulus (word or object) was presented, and participants were asked to indicate if it was old or new.

In blocks 1–3, each training trial consisted of a display of four objects and four pseudowords were played in succession, and 27 such trials were in each block. Each training trial began with the appearance of four objects, which remained visible for the entire trial. After 2 s of initial silence, each word was heard (randomly ordered, duration of 1 s) followed by two additional seconds of silence, for a total duration of 14 s per trial.

After each training period, participants were tested for knowledge of stimuli co-occurrences. One word and one object were presented on each trial, and participants were asked to indicate how many times [0–6] the given word-object pairing had appeared during training. Each of the 18 words and objects appeared in three test trials, for a total of 54 trials, randomly-ordered for each participant. The correct (6-co-occurrence) pairings comprised 18 of the test trials (see diagonal of Figure 1). The remaining 32 trials tested cells in the matrix with 0 co-occurrences (14 trials), 1 (14), 2 (12), 3 (8), and 4 co-occurrences (6 trials).

#### 2.1.3. Procedure

Condition order was fixed, and each participant took part in all four blocks. Block 0 was a three trial training period with three words and objects per trial, followed by a recognition test of every individual stimulus presented, and nine new words and objects. In block 1, the Memory condition, participants were instructed to study individual stimuli for a memory test. However, following the 27 training trials, participants were instead asked to indicate how many times [0–6] each of 54 specific word-object pairings appeared during training. In block 2, participants were asked to track how often each word co-occurred with each object (Count Co-occurrences). After the 27 training trials—which had the same co-occurrence statistics as in block 1, albeit different stimuli—participants were again given test trials asking them to rate the same 54 pairings. Finally, block 3 (Word Meanings) simply instructed participants to learn the meanings of the words, after which they were given cross-situational training (statistically identical to blocks 1 and 2), and again tested on the same 54 pairings.

### 2.2. Results and discussion

In block 0, participants recognized a mean of 96% of the objects and 90% of the words, with a low false alarm rate (8%). In both word and object recognition, every participant was at least 77% accurate. It is notable that memory is imperfect for the stimuli, since many models of cross-situational learning assume that learners can absolutely identify each stimulus, which is evidently not the case.

**Figure 2:**
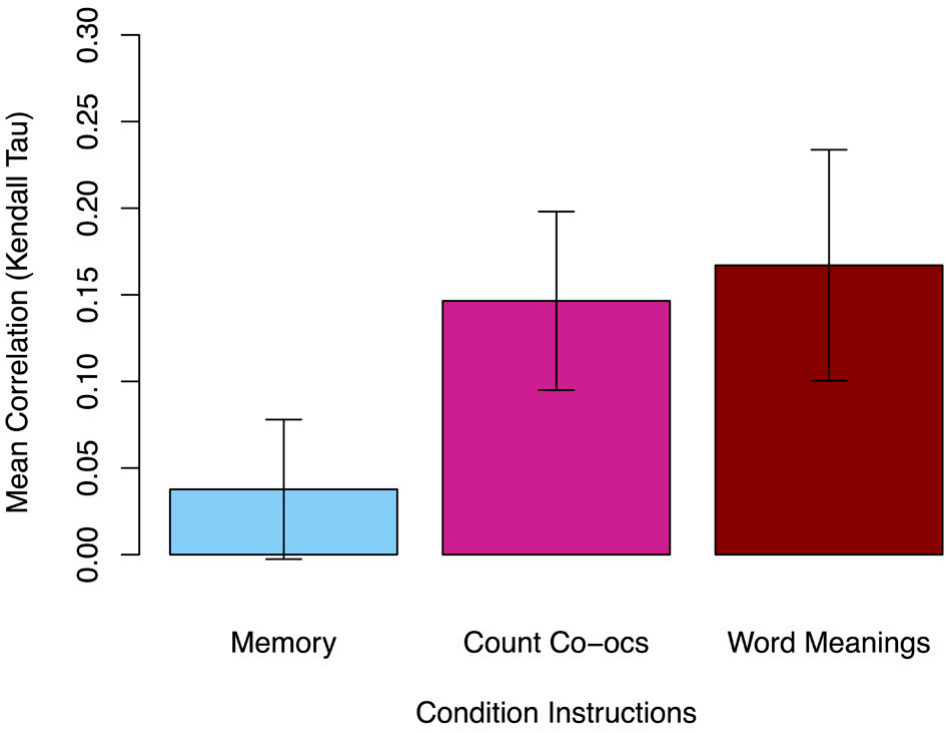
**Mean rank correlation (i.e., Kendall's τ) of participants' responses with the actual pair co-occurrences in Experiment 1.** The count co-occurrence and learn word meanings instructions produced larger correlations than the memory instructions. Error bars show 95% confidence intervals (CI).

To determine how related participants' co-occurrence ratings were to the actual number of times the tested word-object pairings actually appeared together during training, Kendall's rank correlation coefficient (tau) was calculated for each participant's 54 test trials in each condition. The mean tau values for each condition are shown in Figure 2. In block 1, when participants were studying individual words and objects (but not attending to co-occurrences), their responses in the surprise rating task showed a small but significant positive correlation with the actual number of times the presented pairings co-occurred during training [*M* = 0.04, two-tailed *t*_(34)_ = 1.90, *p* = 0.066]. To check this marginal correlation, we bootstrapped bias-corrected and accelerated (BCa; 5000 samples, SE = 0.016) 95% confidence intervals and found they did not overlap with zero: [0.01, 0.07]. In the explicit learning conditions in blocks 2 and 3, when participants were respectively told to track all word-object co-occurrences and to learn the meaning of the words, their ratings were significantly more positively correlated than in block 1 [block 2 *M* = 0.15, paired *t*_(34)_ = 3.82, *p* < 0.001; block 3 *M* = 0.17, paired *t*_(34)_ = 3.86, *p* < 0.001]. Moreover, the strength of correlations in the two explicit conditions is not significantly different [paired *t*_(34)_ = 0.66, *p* > 0.05].

**Figure 3:**
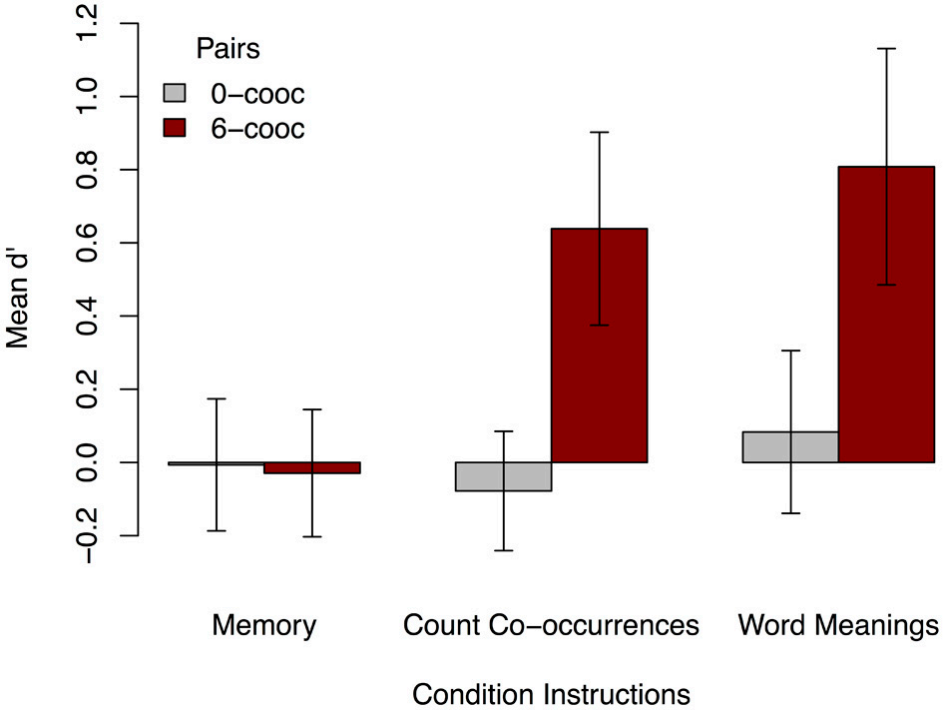
**Mean *d*′ for 0- and 6-co-occurrence word-object pairings in Experiment 1, by instruction condition.** Learners only show significant *d*′ for 6-co-occurrence pairs, and only then under the learn word meanings or count co-occurrences instructions. Error bars show 95% CI.

Positive correlations between ratings and a broad sample of the actual co-occurrence statistics from training indicate that participants are sensitive to arbitrary stimuli co-occurrences when explicitly told to attend to such correspondences. However, one could imagine that the positive correlations could be due largely to knowledge of some particular subsets of the co-occurrences: e.g., perhaps learners are sensitive to words and objects that never co-occurred, and thus rated these pairings very low, and all others high. To examine performance in more detail, we calculated each participant's *d*′ (*d*′^1^) for the most extreme pairings tested in each condition: stimuli that co-occurred 0 or 6 times. Positive *d*′ shows sensitivity resulting from a high hit rate and low false alarm rate. As shown in Figure 3, participants only had significant sensitivity for 6-co-occurrence (“correct”) pairings in the explicit learning conditions [count co-occurrences *M* = 0.64, two-tailed *t*_(34)_ = 4.92, *p* < 0.001; word meanings *M* = 0.81, two-tailed *t*_(34)_ = 5.08, *p* < 0.001].

Two patterns from this study are noteworthy. First, based on both *d*′ analysis and correlation measures, the learning that results from the counting co-occurrences condition and the word learning condition were similar. Although not conclusive, this may suggest that participants in the word learning condition may have used an associative learning strategy based on counting word-object co-occurrences. Second, despite having good recognition memory for individual words and objects that were presented during training, word learning was very poor in the implicit learning condition, as measured both by correlation of their responses with actual pair co-occurrences, and by *d*′ for correct pairings and stimuli pairings that never co-occurred. Nonetheless, the positive correlations found in every condition—although smaller in the implicit condition—show that participants do, on average, absorb some of the stimulus co-occurrences in all conditions. However, this sensitivity is not enough to support implicit word learning in our study, as much stronger correlations are shown when learners are instructed to count co-occurrences or learn word meanings. Under these instructions, participants become sensitive to words and objects that frequently co-occur.

Experiment 2 investigates whether automatic word learning results from a different task, which directs learners' attention to word-object co-occurrences rather than the individual stimuli themselves, as in Experiment 1.

## 3. Experiment 2

Experiment 1 showed that an incidental memory task results in some implicit knowledge of word-referent co-occurrences, but that explicit instructions to learn word-object co-occurrence or to learn word meanings resulted in much greater knowledge. In Experiment 2, we use a different cover task in the implicit learning condition: instead of asking participants to remember individual stimuli for a later memory task, we give participants a signal detection task to carry out during training. In this signal detection task, participants were asked to detect sporadic visual noise added to the presented objects and randomly amplified auditory stimuli, and indicate these occurrences with a keypress. The signal detection task thus directed participants to pay attention to both visual and auditory stimuli simultaneously, but gave no directions to engage in learning of word-object pairings.

### 3.1. Materials and methods

#### 3.1.1. Subjects

Thirty-seven undergraduates at Indiana University received course credit for participating. None had participated in previous cross-situational experiments.

#### 3.1.2. Stimuli

The sets of pseudowords and referents for Experiment 2 were identical to those used in Experiment 1. Training trials were the same as those used in Experiment 1, and had the same co-occurrence statistics (shown in Figure 1). However, on each training trial in blocks 1 and 2, a random number [0–4] of the words were louder than others, and Gaussian pixel noise was momentarily added to a single object *during a word presentation* a random number of times [0–4] each trial. Thus, for 6.3% of audio stimulus presentations during training, that word would be loud and one of the objects would simultaneously have noise added, highlighting a pairing—but only the correct pairing in 25% of these cases. Thus, to perform the cover task perfectly it was necessary to both watch the stimuli for any change, as well as listen for volume changes in the auditory stimuli.

#### 3.1.3. Procedure

In block 1, participants were told that they would be presented with artificial words and objects on a series of slides, on which some words would be louder than the others and some objects would have multicolored speckles (noise). Their task was to quickly press the mouse button each time a loud word or noisy object was presented. However, after the 27 training trials, participants were given a surprise test, and asked to indicate how many times [0–6] each of 54 specific word-object pairings appeared during training. In block 2, participants were asked to track how often each word co-occurred with each object, and were also told to do the same signal detection task during training. Instructions for block 3 asked participants to track word-object co-occurrences without doing the signal detection task, and in block 4 participants were simply told to learn the meanings of the words. The same 54 rating test trials of specific pairings followed the training periods of blocks 2, 3, and 4, though with different stimuli for each block.

### 3.2. Results and discussion

**Figure 4:**
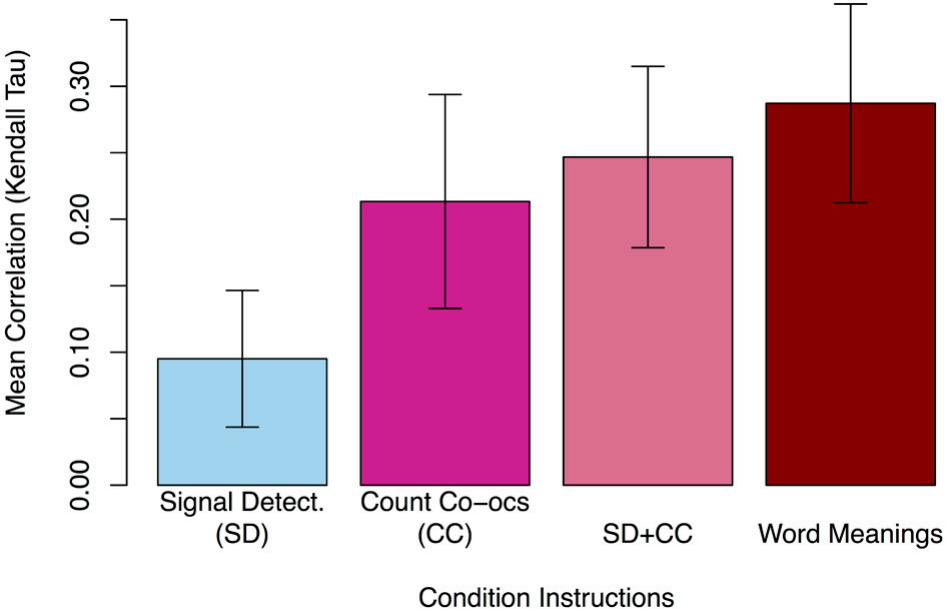
**Mean rank correlation (τ) of each participant's responses with the actual number of pairing co-occurrences in Experiment 2.** Asking participants to learn word meanings produced the highest correlation, followed closely by the joint signal detection and count co-occurrences dual-task condition (SD + CC) and the count co-occurrences task. The signal detection task alone produced a significant but small positive correlation. Error bars show 95% CI.

Experiment 2 used a signal detection (SD) task that required participants to attend to both auditory and visual stimuli, but did not mention that they would need to remember the stimuli later. However, as in Experiment 1, after this first training block participants were given a surprise test for incidental learning. In successive learning conditions, participants were instructed to do both the SD task and to count word-object co-occurrences (SD + CC), to count co-occurrences (with no other task; CC), and finally, to simply learn the meanings of the words (Word Meanings). As in Experiment 1, Kendall's tau was calculated for each participant's 54 test trials in each condition to measure how related their ratings were to the actual number of word-object co-occurrences. As shown in Figure 4, although the SD task resulted in significantly positive correlations (*M* = 0.10, two-tailed *t*_(36)_ = 3.75, *p* < 0.001), the explicit learning conditions showed significantly more correlated responses [CC *M* = 0.21, paired *t*_(36)_ = 3.57, *p* < 0.01; SD + CC *M* = 0.25, paired *t*_(36)_ = 5.12, *p* < 0.001; Word Meanings *M* = 0.29, paired *t*_(36)_ = 4.97, *p* < 0.001]. Thus, as found in Experiment 1, participants show sensitivity to stimuli co-occurrences in every condition, but greater sensitivity in the explicit learning conditions than in the implicit learning condition.

**Figure 5:**
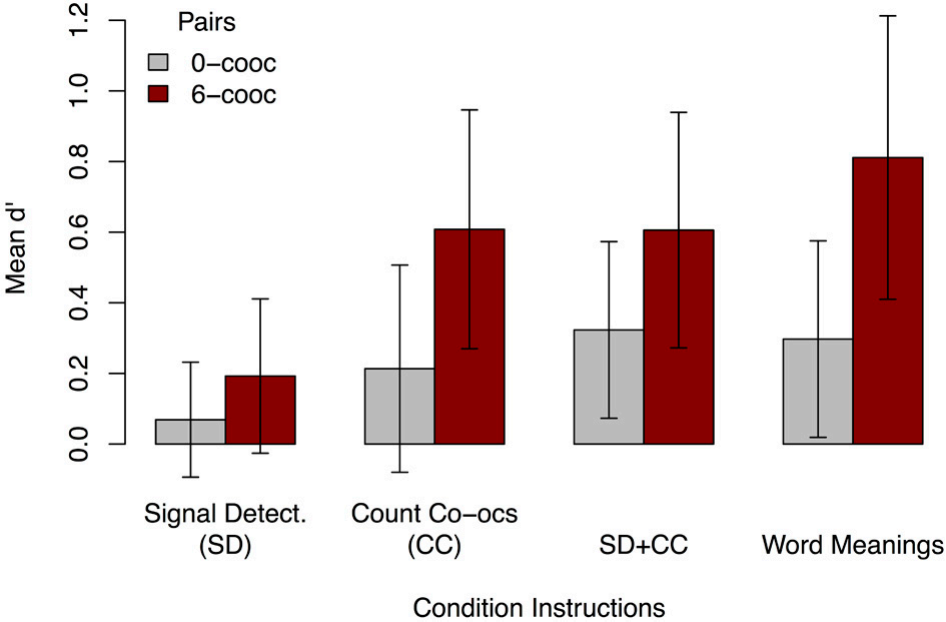
**Mean *d*′ for 0- and 6-co-occurrence word-object pairings in Experiment 2, by condition.** As in Experiment 1, learners were more sensitive to correct (i.e., 6-co-occurrence) pairings than to 0-co-occurrence pairs, although in Experiment 2 they were sensitive to these pairings, except in the signal detection (SD) and count co-occurrences (CC) conditions. The SD condition showed less sensitivity to correct pairings than the other conditions. Error bars show 95% CI.

As in Experiment 1, we calculated *d*′ for maximal and minimal co-occurrence pairings by condition to gain insight into the kind of pairings to which participants in Experiment 2 were sensitive. Figure 5 shows that participants had marginal sensitivity for 6-co-occurrence pairings in the implicit learning condition [SD *M* = 0.19, two-tailed *t*_(36)_ = 1.81, *p* = 0.08, bootstrapped BCa 95% CI = [0.003, 0.411]], but showed significantly greater sensitivity in the explicit conditions [CC *M* = 0.61, paired *t*_(36)_ = 2.97, *p* < 0.01; SD + CC *M* = 0.61, paired *t*_(36)_ = 3.44, *p* = 0.001; word meanings *M* = 0.81, paired *t*_(36)_ = 3.58, *p* < 0.001]. In the explicit conditions, *d*′ for 0-co-occurrence pairings was not significantly positive in the count co-occurrences condition [CC *M* = 0.49, two-tailed *t*_(36)_ = 1.50, *p* = 0.14, bootstrapped BCa 95% CI = [−0.042, 0.511]], but was significantly positive in the dual-task and word meaning conditions [SD + CC *M* = 0.32, two-tailed *t*_(36)_ = 2.66, *p* = 0.01; word meanings *M* = 0.30, two-tailed *t*_(36)_ = 2.20, *p* < 0.05]. In the implicit condition, *d*′ for 0 co-occurrence pairs was not significantly positive [SD *M* = 0.07, two-tailed *t*_(36)_ = 0.87, *p* = 0.39]. Thus, although participants given SD instructions did show some implicit learning of 6-co-occurrence pairings, they were more sensitive to these pairings under explicit instruction.

There are a few intriguing results from this experiment. First, performance in the SD + CC condition was at least as good as CC alone. Thus, participants could handle the two tasks concurrently without hindering performance. We suspected that the signal detection task might encourage participants to attend to both auditory and visual streams simultaneously, perhaps increasing storage of cross-modal associations. Possibly as a result of this focus, in contrast to Experiment 1, participants in Experiment 2 showed significant sensitivity to 0-co-occurrence pairings in the explicit conditions.

Second, word-learning instructions yielded performance as high as found in other explicit instructions (SD + CC and CC). This confirmed our finding from Experiment 1: both counting co-occurrences—as a learner with perfect memory might do—and attempting to learn words result in similar performance in humans, both for correct pairs and for spurious co-occurrences.

## 4. Meta-analysis of explicit learning conditions

In an attempt to unveil the major factors contributing to cross-situational learning, we measured several environmental factors about each item encountered in training, and compared their effects on responses and performance in the learn word meanings and count co-occurrence conditions^2^. We measured contextual diversity (number of distinct words a word is heard with during training, range: 11–13), which is known to influence cross-situational word learning (Kachergis et al., 2009, 2014b). We also measured age of exposure (the trial at which a word first appears, range: 1–6) and context familiarity (the mean number of prior appearances of words heard with a given word, range: 2.94–4.00), which have also been shown to sometimes influence word learning (Fazly et al., 2010; Kachergis et al., 2014b). The final factor investigated is the entropy of each word *w*'s associates (i.e., ∑*_o_* *p*(*o*, *w*)*log*(*p*(*o*, *w*)), where *o* indexes objects), which measures the uncertainty (i.e., dispersion of belief) about *w*'s meaning. Entropy (i.e., uncertainty; range: 3.24–3.52) is an important component of a recent cross-situational learning model which assumes learners are biased to attend to stimuli with uncertain associates (Kachergis et al., 2012a).

In two regressions, these factors^3^ were used to separately predict responses (i.e., the [0–6] ratings) and correctness from the 10,368 test trials from the learn word meanings and count co-occurrences conditions of both experiments. It may be that the differences in instruction result in strategy differences, causing factors to matter in one condition, but not the other. On the other hand, if the same factors influence both conditions, it may be that the same strategy or learning mechanisms are at play in both conditions, regardless of instruction. It is interesting to learn not only which factors predict participants' particular ratings—roughly explaining the rank correlation results presented earlier—but also which factors contributed most to correct responses (i.e., roughly explaining the earlier *d*′ analyses).

First, we fit a linear mixed-effects regression model to predict subjects' responses. Subject was coded as a random effect, and instruction condition was coded as a main effect, with the count co-occurrences condition serving as the intercept. We fit a model with an intercept and all five factors (co-occurrences, contextual diversity, age of exposure, context familiarity, and entropy) as main effects, allowing a possible interaction of condition with each of the factors. This model's coefficients are displayed in Table 1, (AIC = 38,235). For comparison, a null model with only an intercept has AIC = 39,193^4^. As expected given the task, co-occurrences had a significant positive coefficient (0.19). The intercept representing the count co-occurrences condition was significantly positive (2.31), and there was no significant main effect of the word meaning condition (WM; meaning this condition was not different), nor of context familiarity. Age of exposure and contextual diversity had marginally significant positive coefficients (0.03 and 0.15), indicating that later first exposure or appearing with more other items predicts higher responses. Entropy had a significant negative coefficient (−2.05), meaning that higher-entropy words resulted in lower co-occurrence ratings. The only significant interaction was Cond:WM^*^Coocs (0.05), showing that responses in the word meaning condition showed greater influence of co-occurrences (Coocs) than responses in the CC condition. The roughly equivalent response behavior in the two conditions may be because the same basic learning strategies are being applied in both situations, a notion that is reinforced by the significant influence of age of exposure, contextual diversity, and entropy—factors that are known to influence word learning. Next, we investigate how these factors influence the accuracy of the co-occurrence ratings in the two explicit learning conditions (WM and CC).

**Table 1:** **Regression coefficients predicting responses**.

	Coefficient	SE	*t*-value	*p*-value
Intercept (CC)	2.31	0.14	16.94	<0.001
Condition:WM	−0.05	0.04	−1.19	0.24
Coocs	0.19	0.01	20.49	<0.001
Context familiarity	−0.07	0.07	−1.02	0.31
Age of exposure	0.03	0.02	1.85	0.06
Contextual diversity	0.15	0.09	1.66	0.10
Entropy	−2.05	0.93	−2.20	<0.05
Cond:WM^*^Coocs	0.05	0.01	3.59	<0.001
Cond:WM^*^Context familiarity	0.01	0.10	0.07	0.95
Cond:WM^*^Age of exposure	−0.01	0.03	−0.29	0.77
Cond:WM^*^Contextual diversity	−0.17	0.13	−1.34	0.18
Cond:WM^*^Entropy	1.95	1.31	1.48	0.14

We fit a multi-level logistic regression to predict correct responses. Because accuracy (i.e., responding with the exact number of co-occurrences that a given word and object actually coincided) was very low (*M* = 0.20 for the explicit conditions, overall), we defined accuracy more loosely, treating a response within 1 co-occurrence of the actual as correct (e.g., responses of 0, 1, or 2 would be coded as correct for 1 actual co-occurrence). The mean accuracy of explicit conditions for this lenient definition of correctness was 0.44. As in the response model, subject was coded as a random effect, and instruction condition was coded as a main effect, with the count co-occurrences condition serving as the intercept. As before, in addition to the intercept all five factors (co-occurrences, contextual diversity, age of exposure, context familiarity, and entropy) were included as main effects, and a interaction terms for each factor with the word meaning (WM) condition were included.

Table 2 shows the estimated regression coefficients^5^ predicting correctness. The intercept representing the CC condition was significantly negative (−0.37), showing that participants are more likely to be incorrect than correct, and the main effect of the word meaning condition, though estimated to be positive (0.07) was not significant. Greater co-occurrences had a significant negative impact on accuracy (−0.19), showing participants were more accurate for the lower co-occurrence pairings. Significant negative coefficients were also found for age of exposure (−0.08) and contextual diversity (−0.61), showing that later exposure and more diverse contexts—which lead to higher responses in the response regression—produce lower accuracy. context familiarity had a significant positive effect (0.23), indicating that appearing with more familiar items during training is beneficial. Higher entropy, which drove lower responses above, had a significant positive effect (6.73) on accuracy, suggesting that uncertain stimuli were more likely to get accurately low ratings. Finally, the only significant interaction term was a positive effect of the word meaning condition with co-occurrences, showing that high co-occurrence pairs in that condition showed higher accuracy.

**Table 2:** **Regression coefficients predicting correctness**.

	Coefficient	SE	Wald's *z*	*p*−value
Intercept (CC)	−0.37	0.06	−6.60	<0.001
Condition:WM	0.07	0.06	1.27	0.21
Coocs	−0.19	0.01	−14.21	<0.001
Context familiarity	0.23	0.10	2.31	<0.05
Age of exposure	−0.08	0.03	−3.03	<0.01
Contextual diversity	−0.61	0.13	−4.83	<0.001
Entropy	6.73	1.30	5.17	<0.001
Cond:WM^*^Coocs	0.04	0.02	2.12	<0.05
Cond:WM^*^Context familiarity	−0.06	0.14	−0.40	0.69
Cond:WM^*^Age of exposure	0.02	0.04	0.57	0.57
Cond:WM^*^Contextual diversity	0.25	0.18	1.43	0.15
Cond:WM^*^Entropy	−2.95	1.82	−1.62	0.11

Overall, nearly the same factors influenced accuracy as influenced responses—only context familiarity was significant for predicting accuracy, but not for responses. In both regressions, there was little difference between the factors influencing the two explicit conditions, although co-occurrences had more influence on learning word meanings condition than on counting co-occurrences. Finding only small differences, it is tempting to suggest that the strategies and mechanisms operating during these two learning contexts may be similar, but of course our measures may not be sensitive enough. However, since we found the same influences of age of exposure, entropy, and contextual diversity in the response and accuracy regressions for both conditions, and these factors are previously known to influence word learning, we do suggest it is meaningful that instructions to count co-occurrences resulted in similar behavior to that after word learning instructions.

## 5. General discussion

Implicit learning and statistical learning both describe an agent's adaptation to regularities in its environment. We set out to determine whether cross-situational word learning can be accomplished by mere exposure to the same type of training used in intentional settings. In Experiment 1's implicit learning condition, participants attempted to remember individual stimuli. In a surprise test of knowledge for word-object co-occurrences, participants' ratings were correlated with the actual number of co-occurrences, meaning that learners had acquired a rough approximation of the real-world statistics, much like associative models predict. However, a signal detection analysis showed no sensitivity to correct word pairings. We suggest this is because participants' focus on studying individual stimuli prevented much storage of the context (i.e., the other stimuli on the trial). As previously suggested by Fiser (2001), it seems reasonable that deploying attention to the wrong features can inhibit automatic learning. Indeed, in subsequent explicit conditions directing attention to coinciding words and objects, participants showed stronger overall correlations, as well as sensitivity to correct pairings. Using a signal detection task rather than a memory task in the first block, thus encouraging concurrent attention to both words and objects, Experiment 2 asked again whether participants acquire cross-situational co-occurrence statistics automatically. Participants demonstrated some implicit knowledge as in Experiment 1, but also showed some sensitivity for correct word-referent pairs. However, in explicit conditions participants showed greater sensitivity to such frequently co-occurring stimuli, as well as significant knowledge of spurious co-occurrences. Furthermore, we found that participants' learning when instructed to count co-occurrences looks similar to learning under instructions to merely learn words, which we speculate may mean that participants utilize a similar strategy in both conditions. Indeed, in a meta-analysis of both experiments, the same factors (contextual diversity, entropy, and age of exposure) influenced subjects' responses and their accuracy in both of these conditions. By asking participants to perform slightly different tasks with the same input and then comparing their resulting learning, it will be possible to determine which regularities are automatically acquired and which must be explicitly attended or computed.

What do the present results tell us about cross-situational statistical learning? First, they indicate that adults do not only perform the task strategically, and therefore could be using some of the same automatic processes that infants are using. Moreover, the results seem to contradict simple hypothesis-testing mechanisms (e.g., Medina et al., 2011; Trueswell et al., 2013), which would typically not maintain information about spurious co-occurrences, and which may not operate automatically, or in a non-word learning context such as the count co-occurrences condition—let alone the two implicit conditions. However, the results also contradict a purely associative account: learning was greater in explicit conditions than in implicit conditions, suggesting that learning may be in part strategic, or at least modulated by attention. Thus, we may say that cross-situational statistical word learning is neither wholly implicit, nor wholly explicit: some statistics are acquired automatically—likely via basic learning and memory mechanisms—and the learning system indubitably uses this information during explicit study, as well. One proposal for a combined model would make and test explicit hypotheses about word-object mappings, but propose them based on implicitly-tracked co-occurrences (Smith et al., 2011).

The fact that the explicit conditions always produced greater sensitivity for the correct pairings than for pairings that never co-occurred suggests that some mechanism for highlighting stimuli that frequently co-occur is at work. A recent model of cross-situational learning (Kachergis, 2012; Kachergis et al., 2012a) has this sort of attentional bias for already-strong associations, and a competing bias to attend to stimuli with uncertain associates (i.e., high-entropy)—which we found to be predictive of higher accuracy in the explicit experimental conditions presented here. In some situations, such biases in an associative model produce inference-like behavior with quick shifts of attention (Kachergis, 2012). Moreover, this model is better able to capture the shape of individual learning trajectories than a hypothesis-testing model in a multi-block cross-situational learning study (Kachergis et al., 2012b).

In summary, although the implicit learning we observed was inferior to the explicit learning, its presence indicates that knowledge of co-occurrence statistics can be acquired incidentally during cover tasks that require some attention to the audiovisual stimuli. Thus, our results agree with and extend the conclusion of Turk-Browne et al. (2005), which showed that visual statistical learning occurs automatically during a cover task, yet requires sufficient attention to the relevant stimuli in order to proceed. Since implicit learning requires few resources, it can be carried out minute-by-minute, hour-by-hour, and day-by-day. Hence, in the long run, cumulative implicit learning may still play an important role in human language acquisition, building up a vast network of associations in memory that help us direct our attention appropriately when a situation demands it. Overall, our work suggests that neither simple associative models that approximate ideal observers, nor hypothesis-testing models relying on explicit inferences capture both the implicit and intentional aspects of cross-situational word learning. We hope that this work will motivate researchers to consider hybrid models that include both strategic, inference-based mechanisms as well as automatic, associative ones (e.g., Smith et al., 2011). Finally, we believe this work represents a step toward integrating the implicit learning and statistical learning literatures, which share common assumptions and goals, yet both offer some unique observations and perspective.

## Author contributions

George Kachergis conceived the study, and all three authors were involved in experimental design. George Kachergis acquired and analyzed the data, with interpretation help from Chen Yu and Richard M. Shiffrin. George Kachergis drafted the paper, with significant revisions contributed by Chen Yu and Richard M. Shiffrin.

## Funding

This research was supported in part by National Institute of Health Grants R01HD056029 and R01HD074601.

### Conflict of interest statement

The authors declare that the research was conducted in the absence of any commercial or financial relationships that could be construed as a potential conflict of interest.

## Footnotes

^1^For example, hits for 0-co-occurrence pairings are responses of 0, and false alarms are responses of 0 for pairings that co-occurred more than never. *d*′ = *Z*(*p*(hit)) − *Z*(*p*(false alarm)), where *Z* is the inverse of the cumulative Gaussian distribution. Hits for 6-co-occurrence pairings are responses of 6, although counting 5 (which never occurs spuriously) and 6 as a hit yields nearly identical results.

^2^The trials from the two implicit conditions and the dual-task condition (SD + CC) were excluded both because we are interested in which factors influence explicit attempts to learn, and because the two implicit tasks may not be comparable.

^3^Regressors were re-centered at 0 to limit the effects of collinearity in the interaction terms.

^4^Akaike's information criterion (AIC) is used for model selection; lower is better.

^5^The coefficient estimates a predictor's effect size in terms of log odds ratio.
